# The neurological implications of metacognition

**DOI:** 10.3389/fpsyg.2026.1756611

**Published:** 2026-02-24

**Authors:** Philip R. Hulbig

**Affiliations:** Curry College, Program for the Advancement of Learning, Milton, MA, United States

**Keywords:** clinical interventions, metacognition, neural reorganization, neuroplasticity, neuroscience, prefrontal cortex

## Abstract

This review synthesizes current understanding of metacognitive processes across neuroscientific, clinical, and educational domains. Metacognition, defined broadly to encompass knowledge/awareness, and monitoring/regulation of one’s cognitive processes spans both neurological and psychological domains. This quality holds significant implications for human development. Neuroimaging evidence suggests that metacognition relies on distributed networks spanning prefrontal-parietal circuits, with connections to the anterior cingulate and hippocampus. These regions demonstrate experience-dependent structural changes, as well as regulate the cognitive processes that drive neuroplastic behavior. The bifurcated model of metacognition, which distinguishes between metacognitive knowledge and regulation, represent an embodied perspective of a prediction based problem-solving framework that can be used to inform the development of clinical and educational interventions seeking to support neurodiversity. Evidence suggests that targeted intensive cognitive training may produce neural changes through well-characterized neuroplastic mechanisms. However, establishing causal links between metacognitive training specifically and structural brain reorganization requires additional research with appropriate neuroimaging protocols and control conditions. These converging lines of evidence establish a metacognitive problem-solving axis encompassing neural, cognitive, and behavioral functioning as the primary mechanism for controlled psychobiological reorganization. By focusing an individual’s powers of problem solving upon their own development and understanding of their own problem-solving process, clinical interventions can be developed that are less coercive and more supportive of individual neurodiversity. The neurological implications of metacognition suggest that individuals can be supported in developing the optimal environments, procedures, and pedagogies to improve their learning and development, in turn affecting their underlying neurological architecture.

## 1 Introduction

### 1.1 The problem of self-development

Human beings possess a remarkable capacity for self-development that distinguishes them from other species. This capacity for self-development is underpinned by a neurological process described as metacognitive awareness, which enables individuals to monitor their cognitive processes, evaluate their understanding, and strategically modify their approaches to learning and problem-solving. This ability to regulate one’s own thinking has emerged as perhaps the most critical skill for adaptive functioning in highly diverse modern environments (Fleming and Dolan, 2012).

Metacognition encompasses a related set of functions including self-monitoring, judgment of learning, feeling of knowing, error detection, confidence ratings, and strategic regulation (Flavell, 1979; Nelson and Narens, 1990). These processes operate at different levels of complexity and engage partially dissociable neural systems. This heterogeneity presents both conceptual and methodological challenges when attempting to characterize the neural substrates and clinical applications of metacognition. This review synthesizes current understanding across three interconnected domains: neural substrates of metacognitive processing, neuroplastic changes through training, and clinical/educational applications.

### 1.2 Theoretical origins and core concepts

The formal study of metacognition began with Flavell (1979), p. 906, who defined it as “knowledge and cognition about cognitive phenomena.” Wells and Purdon (1999) described metacognition as thinking about thinking, capturing the recursive nature of metacognitive awareness. Nelson and Narens (1990, 1996) expanded on the conceptualization as an interaction between object-level subjective cognitive operations focused on the external world and task execution, and an internally focused meta-level of symbolic cognitive operations that monitor the abstract relationships revealed by the processes of the object level. This framework has guided numerous experimental investigations, particularly in metamemory research building on Hart’s (1965) foundational work on feeling-of-knowing judgments.

Bifurcated models of the mind have existed at least since the first discovery that the physical brain itself was divided into two hemispheres. However, modern neuroscience seems to be converging on a two-dimensional model of the mind with tremendously flexible problem-solving capacity. With one dimension being focused on building perceptions based on prediction (Seth, 2021) and the other dimension being one which reflects on those predictions and error-corrects to improve the quality of future predictions (Deutsch, 2011). This model can be thought of as a two-step problem-solving paradigm that underpins all problem-solving, and can be applied therapeutically and pedagogically to support human behavior and development (Hulbig, 2024).

## 2 Methods

### 2.1 Literature search strategy

We conducted a comprehensive narrative review of the metacognition and neuroplasticity literature. Primary sources included PubMed, Web of Science, and PsycINFO databases, searching combinations of terms including: “metacognition,” “metacognitive,” “neuroplasticity,” “brain plasticity,” “prefrontal cortex,” “anterior cingulate,” “hippocampus,” “BDNF,” combined with “intervention,” “training,” “therapy,” “education,” “clinical,” and “rehabilitation.”

We prioritized peer-reviewed research published between 2000 and 2025, with emphasis on neuroimaging studies, randomized controlled trials, meta-analyses, and systematic reviews. Additional sources were identified through citation tracking of seminal works including Flavell (1979), Nelson and Narens (1990, 1996), Fleming and colleagues’ neuroimaging research, and theoretical frameworks by Lysaker et al. (2018), Morris and Mograbi (2013), and Crosson et al. (1989).

**Figure 1:**
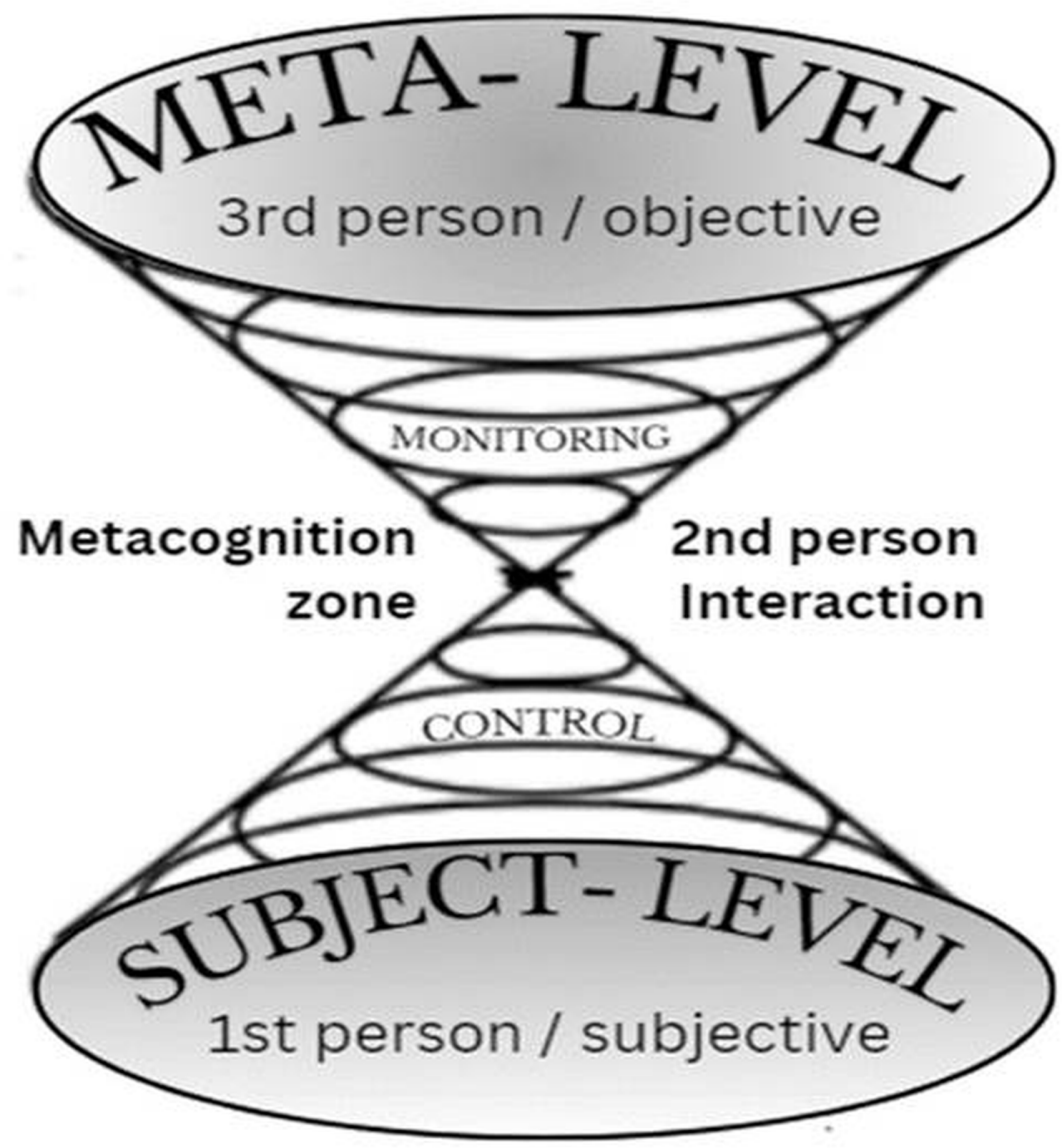
Two modes of metacognitive consciousness and their relationship to communication styles. This figure illustrates Nelson and Narens (1990) influential bifurcated model of metacognition, which distinguishes between object-level cognitive processes (focused on task execution and interaction with the external world) and meta-level cognitive processes (focused on monitoring and controlling object-level operations). The visualization has been adapted to show the relationship between these metacognitive levels and communication patterns. Object-level processes correspond to first-person subjective experience and language (“I perceive,” “I think”), while meta-level monitoring corresponds to second-person observational perspective (“You are doing X”), and meta-level control corresponds to third-person objective analysis (“One should do Y”). This integration demonstrates how metacognitive awareness operates across different levels of self-reference and provides a framework for understanding how individuals monitor and regulate their own cognitive processes through various modes of internal dialogue and self-reflection. The bidirectional arrows indicate the dynamic flow of information between levels, with the meta-level both monitoring object-level activity and exerting control to modify subsequent processing.

We also incorporated relevant neuroscience research on neuroplasticity mechanisms, particularly studies examining experience-dependent brain changes and molecular mechanisms of synaptic plasticity. Given the narrative format, we synthesized findings thematically rather than conducting quantitative meta-analysis (see Figure 1).

### 2.2 Inclusion criteria

Studies were included if they: (1) examined neural substrates of metacognitive processes through neuroimaging or lesion studies, (2) investigated neural changes resulting from cognitive or metacognitive training interventions, (3) reported clinical or educational interventions explicitly targeting metacognitive processes with measurable outcomes, or (4) explored molecular mechanisms (particularly BDNF pathways) underlying experience-dependent plasticity relevant to metacognitive enhancement.

For clinical studies, we recorded patient population characteristics including neurological conditions (stroke, traumatic brain injury), psychiatric diagnoses (schizophrenia, depression, anxiety disorders), and learning differences. Studies were excluded if they examined metacognition purely theoretically without empirical evidence, lacked peer review, or provided insufficient methodological detail.

Given the narrative review format, this synthesis does not claim to be exhaustive. The limitations inherent in narrative synthesis may affect the comprehensiveness and balance of findings presented.

## 3 Results

### 3.1 Neural substrates of metacognition

Neuroimaging research has identified distributed brain networks supporting metacognitive processes, though considerable heterogeneity exists depending on which specific metacognitive functions are examined.

#### 3.1.1 Prefrontal cortex

Meta-analytic evidence (Vaccaro and Fleming, 2018) analyzing 47 neuroimaging studies identified consistent activation in the medial and lateral prefrontal cortex during metacognitive judgments. The right rostrolateral prefrontal cortex (rlPFC) demonstrates particular importance, showing increased functional connectivity with both contralateral prefrontal regions and task-specific sensory areas during metacognitive reports (Fleming et al., 2012).

Individual differences in metacognitive ability correlate with gray matter volume in the anterior prefrontal cortex (Fleming et al., 2010). Recent transcranial magnetic stimulation studies provide causal evidence for prefrontal involvement: disruption of lateral PFC impairs metacognitive accuracy while preserving first-order task performance (Lapate et al., 2020; Miyamoto et al., 2021). Importantly, different prefrontal subregions contribute to distinct aspects of metacognition (Schurz and Perner, 2015), with more recent work showing the frontal lobe processes difficult metacognitive judgments by reading out performance information from first-order task regions (Soutschek and Mattes, 2025).

#### 3.1.2 Anterior cingulate cortex

The dorsal anterior cingulate cortex (dACC) plays a critical role in monitoring cognitive processes and detecting conflicts. Carter et al. (1998) demonstrated that the ACC activates not only during errors but also during correct responses under high response competition, indicating a monitoring function rather than simple error detection. Subsequent research identified a dACC network specifically involved in metacognitive uncertainty (Qiu et al., 2018a,b; Su et al., 2022).

Meta-analytic evidence confirms that dACC and bilateral anterior insula function as an integrated network hub for self-regulation (Zhang and Peng, 2023). Brain stimulation studies using transcranial direct current stimulation support the causal role of dACC in metacognitive bias (Soutschek and Mattes, 2025). Notably, the ACC demonstrates structural plasticity, with morphological changes related to learning and practice (Newman and McGaughy, 2011).

#### 3.1.3 Parietal cortex

Posterior parietal regions, particularly the precuneus and inferior parietal lobule, consistently activate during metacognitive judgments across domains (Vaccaro and Fleming, 2018). The parietal cortex may accumulate sensory evidence and confidence-related signals that inform metacognitive decisions. Effective connectivity analyses suggest information flow from posterior to anterior regions during confidence judgments, consistent with hierarchical processing models.

#### 3.1.4 Hippocampus

While traditionally viewed as a memory structure, the hippocampus contributes to metacognitive monitoring across domains. Connection strength between hippocampus and precuneus correlates with confidence ratings. Hippocampal microstructure relates to metacognitive ability even in perceptual tasks (Allen et al., 2017).

Ren et al. (2018) demonstrated that effective connectivity from anterior hippocampus to precuneus during stimulus viewing predicts subsequent metacognitive confidence in voluntary recall. Intracranial recordings reveal distinct communication patterns between hippocampus and precuneus during metacognition (Chen et al., 2024). Resting-state connectivity studies confirm that increased hippocampal-precuneus connectivity associates with metamemory ability (Ye et al., 2019).

These findings collectively suggest that metacognitive processes emerge from interactions across distributed networks rather than from discrete localized regions. The specific network configurations engaged likely depend on task domain (perceptual, memory, problem-solving), cognitive load, and individual differences in metacognitive skill.

### 3.2 Metacognitive deficits in neurological conditions

Understanding metacognitive impairments across clinical populations provides crucial insights into the neural basis of self-awareness and the potential for therapeutic intervention. This section first provides an overview of metacognitive deficits across conditions before examining specific illustrative cases.

#### 3.2.1 Overview of metacognitive deficits across populations

Metacognitive impairments manifest across a spectrum of neurological and psychiatric conditions, with deficits varying in severity, domain specificity, and amenability to intervention (Prigatano and Altman, 1990; Agnew and Morris, 1998).

In traumatic brain injury (TBI), metacognitive deficits are common and predict functional outcomes more strongly than objective cognitive impairments (Ownsworth et al., 2019). A meta-analysis of 22 studies found that better metacognitive knowledge following acquired brain injury correlated with improved quality of life, family integration, and work outcomes. These deficits span multiple awareness levels, with patients often demonstrating relatively preserved intellectual awareness while showing marked impairments in emergent and anticipatory awareness.

In schizophrenia, metacognitive deficits represent a core feature affecting multiple domains including self-reflectivity, understanding of others’ minds, and integrated sense of identity (Lysaker et al., 2018). These deficits correlate with negative symptoms, functional outcomes, and subjective recovery more strongly than do neurocognitive deficits alone. Neuroimaging studies reveal associations between metacognitive capacity and prefrontal-temporal connectivity (Sapara et al., 2014).

Depression and anxiety disorders show characteristic patterns of metacognitive dysfunction, particularly in the form of maladaptive metacognitive beliefs, worry and rumination. These beliefs include positive beliefs about worry (“worrying helps me cope”) and negative beliefs about uncontrollability (“my worrying could make me go mad”). Such beliefs maintain cognitive-attentional syndrome patterns including repetitive negative thinking, threat monitoring, and maladaptive coping strategies (Wells, 2009).

#### 3.2.2 Anosognosia: complete lack of deficit awareness

Anosognosia represents the most dramatic example of metacognitive impairment, causing a complete lack of awareness of neurological deficits. Occurring in 10–18% of acute stroke patients with hemiparesis (Prigatano and Schacter, 1991), anosognosia demonstrates how focal brain damage can eliminate self-awareness while preserving other cognitive functions.

Neuroanatomical studies consistently associate anosognosia with right hemisphere lesions affecting parietal lobe, temporoparietal cortex, insular cortex, and thalamus (Orfei et al., 2010). These regions appear critical for integrating sensory, motor, and cognitive information into coherent self-awareness. The dissociation between preserved motor planning and absent awareness of paresis in anosognosia patients suggests that self-monitoring mechanisms operate independently from action systems.

The Cognitive Awareness Model (Morris and Mograbi, 2013) proposes that anosognosia results from failures in comparator mechanisms that normally detect mismatches between intended and actual performance, potentially modulated by emotional/motivational factors that make awareness psychologically intolerable.

#### 3.2.3 Wernicke’s aphasia: domain-specific metacognitive impairment

Left hemisphere damage affecting temporal language areas produces Wernicke’s aphasia leading to fluent but often semantically empty speech with severely impaired comprehension. Many patients show limited awareness of their communication failures, providing insight into domain-specific metacognitive mechanisms for language monitoring.

Recent therapeutic research demonstrates the potential for intervention even in severe cases. Raymer et al. (2024) tested a metacognitive intervention in two participants with severe Wernicke’s aphasia, finding improvements in naming and discourse skills. This suggests that metacognitive awareness can be partially restored through targeted training even when language processing is severely compromised. The mechanisms likely involve engaging preserved monitoring systems and establishing compensatory strategies for error detection.

#### 3.2.4 Alzheimer’s disease and mild cognitive impairment

Progressive neurodegenerative conditions produce evolving metacognitive deficits with characteristic temporal patterns. In mild cognitive impairment (MCI) and early Alzheimer’s disease, anosognosia frequency ranges from 20 to 30% (Orfei et al., 2010), with severity correlating with medial temporal and prefrontal atrophy.

Interestingly, metamemory judgments in early stages can show relative preservation despite objective memory decline, suggesting that monitoring mechanisms can operate partially independently from the cognitive functions they monitor. As disease progresses, metacognitive deficits typically worsen, affecting both monitoring accuracy and strategic regulation.

#### 3.2.5 Measurement considerations

Assessment of metacognitive function requires multiple complementary approaches, each capturing different aspects of metacognitive capacity:

1. Questionnaire discrepancy methods: comparing self-ratings of ability with performance measures or informant reports provides indices of awareness accuracy. The Patient Competency Rating Scale (Prigatano and Altman, 1990) exemplifies this approach, quantifying discrepancies between patient and clinician ratings across functional domains.
2. Performance-based paradigms: confidence ratings collected during cognitive tasks enable calculation of metacognitive sensitivity (calibration between confidence and accuracy) and metacognitive bias (overall confidence level). Signal detection theory approaches formalize these measurements (Fleming and Lau, 2014).
3. Error awareness tasks: procedures requiring explicit error detection or correction assess online monitoring capabilities. The Awareness Questionnaire (Agnew and Morris, 1998) systematically assesses error awareness across task contexts.
4. Structured interviews: the Metacognition Assessment Scale (Lysaker et al., 2018) derived from open-ended interviews evaluates self-reflectivity, understanding of others’ minds, decentration, and mastery through detailed qualitative assessment.
5. Neuroimaging Biomarkers: Structural and functional connectivity patterns in prefrontal-parietal networks provide potential neural markers of metacognitive capacity, though these require further validation for clinical application.

No single measure captures all dimensions of metacognitive function. Comprehensive assessment requires selecting methods matched to the specific clinical question, cognitive domain of interest, and practical constraints of the assessment context (Toglia and Kirk, 2000).

### 3.3 Neuroplasticity and metacognitive enhancement

Evidence that intensive cognitive training produces measurable structural brain changes provides a foundation for hypothesizing that metacognitive interventions might engage similar mechanisms. However, establishing this connection requires careful consideration of what specific training components drive neural reorganization.

The landmark London taxi driver studies (Maguire et al., 2000; Maguire et al., 2006) demonstrate human hippocampal plasticity but require careful interpretation regarding metacognition. Taxi drivers showed significantly larger posterior hippocampi compared to controls, with anterior regions showing opposite patterns. Critically, a follow-up comparing taxi drivers to bus drivers who were matched for driving experience and stress but who followed fixed routes rather than having to constantly access memory found hippocampal changes only in taxi drivers.

#### 3.3.1 What drives the neural changes?

The taxi driver studies demonstrate that intensive spatial learning engaging flexible navigation produces measurable neural reorganization. While not involved in explicit or direct metacognitive training, successful navigation planning and execution of the routines required to develop the skill involves metacognitive processes such as monitoring one’s confidence about route knowledge, regulating search strategies, evaluating whether a route selection was optimal, an various self regulatory functions tied to metacognitive monitoring.

These studies provide proof-of-principle that:

1. Adult human brains can undergo substantial structural reorganization
2. Such reorganization occurs in regions (hippocampus) relevant to metacognition
3. Changes correlate with specific trained skills
4. Reorganization involves not yet well understood trade-offs, with taxi drivers showing reduced ability to acquire new visuospatial information

Whether metacognitive training specifically (targeting monitoring and regulation processes) produces comparable neural changes remains an open empirical question, but the involvement of metacognitive processes in coordinating the behaviors necessary to produce meaningful morphological change in specific brain regions to achieve specific cognitive function is clear.

### 3.4 Molecular mechanisms supporting neural plasticity

Understanding molecular mechanisms underlying experience-dependent plasticity provides a biological foundation for hypothesizing how interventions might support neural reorganization. Brain-derived neurotrophic factor (BDNF) represents a key mediator of neuroplasticity, whose specific connection to metacognitive processes requires further investigation.

#### 3.4.1 BDNF and exercise-induced neuroplasticity

Exercise promotes *β*-hydroxybutyrate production, which modulates histone deacetylases (HDAC2/HDAC3) to increase BDNF expression, supporting neurogenesis, synaptic plasticity, and cognitive function (Seilman, 2016). Meta-analysis of 22 studies with 552 participants showed that high-intensity exercise significantly increases peripheral BDNF compared to light exercise (effect size = 0.78, *p* < 0.001) (Fernández-Rodríguez et al., 2022). Importantly, these acute exercise effects persist in older adults (Nilsson et al., 2020).

#### 3.4.2 Meditation and mindfulness

Mindfulness practices explicitly cultivate metacognitive awareness—observing one’s thoughts and mental states without judgment. Research shows meditation produces measurable neural changes including increased cortical thickness and enhanced connectivity (Calderone et al., 2024). Magnetoencephalography studies of long-term Vipassana meditators reveal significantly higher resting-state hippocampal connectivity (theta band, *p* = 0.009) compared to controls (Martorano et al., 2019), potentially protecting against age-related cognitive decline.

#### 3.4.3 Bridging to metacognition

While exercise and meditation engage neuroplastic mechanisms, the specific causal pathway from these interventions through metacognitive enhancement to structural brain changes remains incompletely characterized. Several potential mechanisms warrant investigation:

1. Indirect pathway: exercise → BDNF increase → general cognitive enhancement → improved capacity for metacognitive monitoring/regulation
2. Attentional pathway: meditation → enhanced attention regulation → improved metacognitive monitoring → strategic skill development engaging plasticity
3. Combined effects: physical and metacognitive training together might produce additive benefits through complementary mechanisms (Cefis et al., 2023)

Current evidence supports these interventions as neuroplasticity-promoting approaches, but establishing direct causal links to metacognitive enhancement specifically requires studies measuring metacognitive outcomes alongside neural changes with appropriate experimental controls.

### 3.5 Clinical applications: metacognitive therapeutic approaches

Two major therapeutic frameworks explicitly target metacognitive processes, representing distinct conceptualizations of metacognition and employing different intervention strategies.

#### 3.5.1 Metacognitive therapy (MCT)

Developed by Wells (2009) and Wells and Purdon (1999), MCT focuses on modifying metacognitive beliefs and processes maintaining psychological distress. Unlike cognitive-behavioral therapy (CBT), which targets thought content, MCT addresses how individuals respond to thoughts, targeting the cognitive-attentional syndrome. These are patterns of worry, rumination, and threat monitoring driven by metacognitive beliefs.

Randomized controlled trials provide compelling evidence for efficacy. The largest trial comparing MCT to CBT in 174 adults with major depression (Callesen et al., 2020) found MCT demonstrated superior outcomes: recovery rates of 74% versus 52% for CBT at post-treatment (odds ratio = 2.42, *p* = 0.014), with significant differences on the Beck Depression Inventory II favoring MCT [−5.49, 95% CI (−8.90 to −2.08), *p* = 0.002]. Benefits persisted at 6-month follow-up (74% vs. 56% recovery), with MCT requiring fewer sessions on average (5.5 vs. 6.7 sessions). Similar patterns emerge for generalized anxiety disorder (Nordahl et al., 2018).

#### 3.5.2 Metacognitive reflection and insight therapy (MERIT)

Developed by Lysaker et al. (2018) and Lysaker et al. (2019) within the Integrative Model of Metacognition, MERIT targets metacognitive capacity as a spectrum of abilities for forming integrated representations of self, others, and mental states. Primarily developed for schizophrenia, MERIT emphasizes narrative development, perspective-taking, and integration of fragmented self-experiences.

Research demonstrates that metacognitive capacity predicts functional outcomes in schizophrenia independent of neurocognition (Lysaker et al., 2018). MERIT aims to enhance: (1) self-reflectivity (recognizing and labeling internal states), (2) awareness of others’ mental states, (3) decentration (recognizing mental states are interpretations), and (4) mastery (using metacognitive knowledge for problem-solving).

Preliminary trials show promise (Lysaker et al., 2018), suggesting moderate effect sizes for functional outcomes. Neuroimaging studies link metacognitive capacity improvements with changes in prefrontal-temporal connectivity (Sapara et al., 2014), though dedicated neuroimaging studies of MERIT specifically are needed.

#### 3.5.3 Neuroplasticity implications

While both approaches demonstrate clinical efficacy, important limitations exist regarding neuroplastic mechanisms. Most MCT trials have not included neuroimaging protocols, so claims about engaging neuroplastic mechanisms remain speculative. The sustained benefits observed in follow-up studies (Callesen et al., 2020) are consistent with but do not prove lasting neural reorganization.

Recent studies examining related metacognitive interventions provide suggestive evidence. Shan et al. (2021) found that 8 weeks of metacognitive training in schizophrenia produced increased regional brain homogeneity across prefrontal and limbic regions on fMRI. However, these studies typically lack active control conditions that would distinguish specific metacognitive training effects from general therapeutic engagement or attention effects.

Future research incorporating neuroimaging into RCTs of MCT and MERIT, with appropriate control conditions, would significantly advance understanding of whether and how these therapeutic approaches engage neuroplastic mechanisms.

### 3.6 Metacognitive interventions in educational settings

Extensive meta-analytic evidence demonstrates associations between metacognitive strategies and academic achievement. Analysis of 147 studies involving 698,096 participants found significant positive correlations between metacognitive strategies and mathematics achievement [*r* = 0.32, 95% CI (0.30, 0.34)] (Xie et al., 2024). The Education Endowment Foundation reports that metacognitive interventions can add up to 7 months of additional learning progress, making them among the most effective educational strategies available. Effects appear particularly beneficial for students with learning differences.

#### 3.6.1 Implementation components

Effective metacognitive educational interventions typically include:

1. Explicit strategy instruction: teaching students specific strategies for planning, monitoring, and evaluating their learning, tailored to particular academic domains
2. Self-questioning protocols: training students to ask themselves questions that prompt metacognitive monitoring (“Do I understand this? What strategy should I try?”)
3. Think-aloud procedures: having students verbalize their thinking processes during problem-solving, making metacognitive processes explicit
4. Metacognitive Scaffolding: Providing structured support that gradually transfers responsibility for monitoring and regulation from teacher to student

Research by Rueda et al. (2018) demonstrated that children receiving metacognitive scaffolding alongside executive attention training showed not only larger skill gains but also significant increases in electrophysiological indices of conflict processing. Critically, these neural changes directly predicted cognitive improvements, suggesting that metacognitive scaffolding enhanced the effectiveness of cognitive training by engaging monitoring and regulation processes.

#### 3.6.2 Conceptual framework

Many strategies identified as metacognitive in educational contexts are actually domain-general problem-solving strategies applied to the problem of self-development (Hulbig, 2023, 2024). This reframes metacognitive skill development as teaching students to conceptualize their learning as a problem requiring systematic problem-solving: defining learning goals, selecting and implementing strategies, monitoring progress, and adjusting approaches based on feedback.

Teachers and mentors can support students in developing these skills by making the problem-solving framework explicit, providing models of expert metacognitive processing, and creating opportunities for deliberate practice with feedback. This approach may be particularly valuable for students with learning differences who face more complex learning challenges requiring systematic self-regulation.

## 4 Discussion

### 4.1 Integration of evidence: current understanding

This review synthesizes evidence across neural, clinical, and educational domains regarding metacognitive processes. Several patterns emerge from this synthesis, highlighting the gaps remain in our understanding.

#### 4.1.1 Neural substrates

Converging evidence from neuroimaging, lesion studies, and brain stimulation research indicates that metacognitive processes rely on distributed networks involving prefrontal cortex (monitoring and control), anterior cingulate (conflict detection and uncertainty), parietal cortex (evidence accumulation and confidence), and hippocampus (particularly for memory-related metacognition). However, substantial heterogeneity exists depending on which specific metacognitive functions are examined, task domain, and individual differences.

Meta-analyses (Vaccaro and Fleming, 2018) provide the most reliable characterization of consistent activation patterns, but show variability reflecting the multifaceted nature of metacognition. Different theoretical frameworks (Fleming’s signal-detection approaches, Lysaker’s integrative model, Wells’ metacognitive therapy framework) emphasize different components of metacognitive function and may engage partially dissociable neural systems.

#### 4.1.2 Clinical applications

Multiple therapeutic approaches targeting metacognitive processes show efficacy across various conditions. MCT demonstrates superior outcomes compared to CBT for depression and anxiety, with benefits maintained at follow-up (Callesen et al., 2020; Nordahl et al., 2018). MERIT shows promise for schizophrenia, where metacognitive capacity predicts functional outcomes beyond neurocognition (Lysaker et al., 2018). Metacognitive interventions for TBI improve awareness and functional outcomes (Ownsworth et al., 2019).

The sustained nature of these clinical benefits raises questions about underlying mechanisms. Are improvements maintained through continued application of learned strategies, through neural reorganization making metacognitive processing more efficient, or through both? Most clinical trials have not included neuroimaging, leaving these questions unresolved.

#### 4.1.3 Educational applications

Meta-analyses consistently show positive associations between metacognitive strategy instruction and academic achievement (Xie et al., 2024), with particularly strong effects for students with learning differences. The magnitude of effects (up to 7 months additional progress) suggests educationally meaningful benefits. Neural studies combining metacognitive scaffolding with attention training show both behavioral improvements and enhanced electrophysiological indices of cognitive processing (Rueda et al., 2018).

#### 4.1.4 Neuroplasticity connections

Evidence that intensive training produces structural brain changes (Maguire et al., 2000, 2006) and that regions critical for metacognition demonstrate experience-dependent plasticity (Newman and McGaughy, 2011) suggests potential mechanisms. Molecular mediators of plasticity including BDNF can be enhanced through exercise and meditation (Fernández-Rodríguez et al., 2022; Calderone et al., 2024), interventions that may complement metacognitive training.

However, direct evidence specifically linking metacognitive training to structural neural reorganization remains limited. Most studies showing neural changes following training involve intensive cognitive tasks that likely engage metacognitive processes but did not explicitly train metacognitive skills. Establishing causal relationships requires studies that: (1) use explicit metacognitive training protocols, (2) include appropriate control conditions, (3) measure both metacognitive outcomes and neural changes, and (4) employ longitudinal designs tracking the time course of change.

### 4.2 Theoretical integration: metacognition as neural learning mechanism

A central theoretical question concerns how metacognitive processes relate to neuroplasticity mechanistically. Several conceptual frameworks suggest potential links:

#### 4.2.1 Prediction-error framework

Contemporary neuroscience increasingly conceptualizes brain function as hierarchical predictive processing (Seth, 2021). Lower levels generate predictions about sensory input; higher levels monitor prediction errors and update models accordingly. Metacognition can be understood as explicit awareness of this prediction-error process—the ability to consciously detect mismatches between expectations and outcomes and systematically adjust strategies.

**Figure 2:**
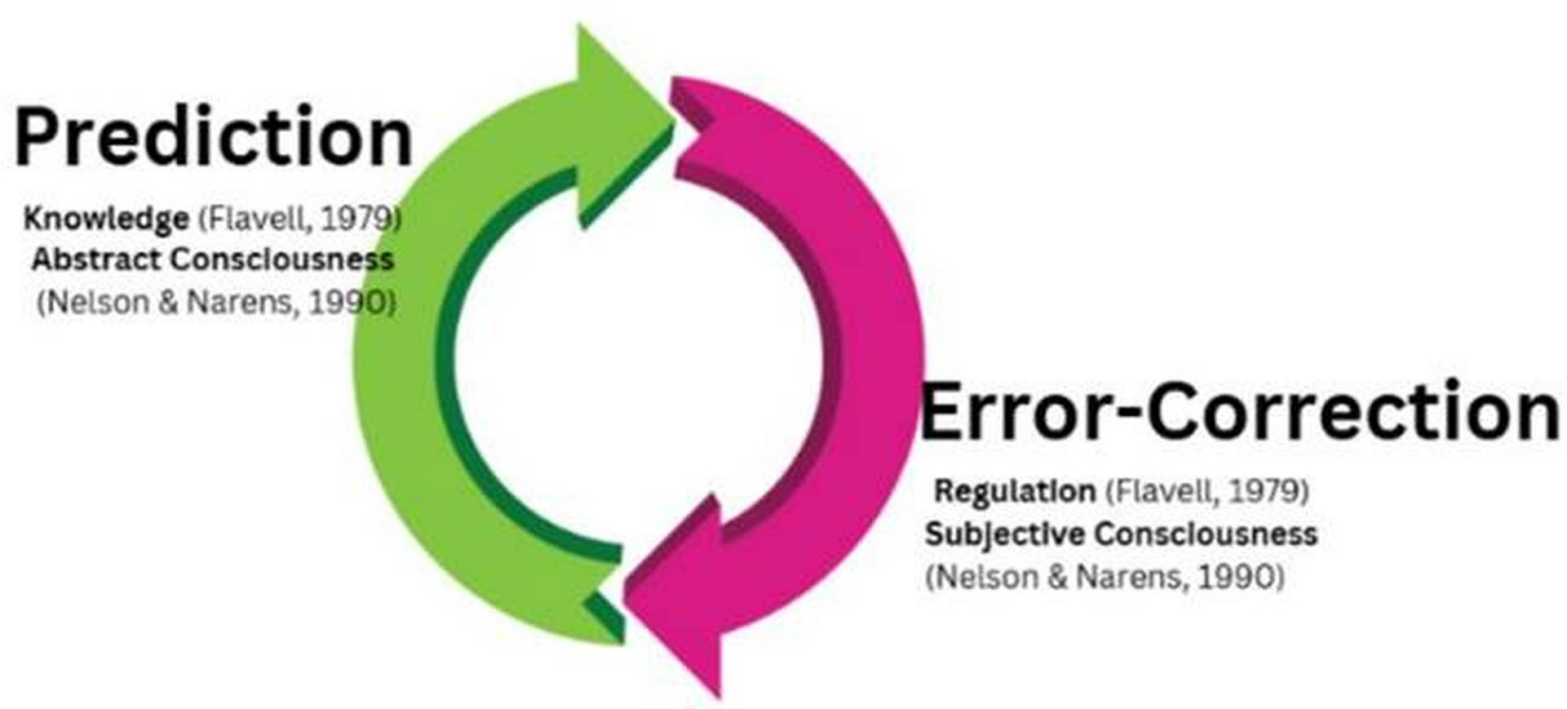
Integration of two-step problem-solving with bifurcated metacognitive models. This original figure synthesizes contemporary neuroscientific understanding of metacognition with classical theoretical models, demonstrating how modern two-dimensional models of mind align with earlier bifurcated conceptions of metacognitive consciousness. The visualization shows the convergence of multiple theoretical perspectives: (1) The predictive processing framework (Seth, 2021) where one dimension focuses on building perceptions based on prediction, (2) the error-correction dimension (Deutsch, 2011) that reflects on predictions and adjusts to improve future accuracy, and (3) the classical metacognitive knowledge-regulation distinction. The figure illustrates how these seemingly disparate frameworks describe the same fundamental two-step problem-solving process: an object-level phase of perception/prediction/action and a meta-level phase of monitoring/evaluation/adjustment. This integration provides a unified theoretical foundation demonstrating that metacognitive processes represent a general problem-solving paradigm applicable across cognitive, therapeutic, and educational domains. The overlapping elements emphasize that contemporary neuroscience is converging on conceptual models that have roots in earlier metacognitive theory, suggesting a fundamental architecture of self-reflective consciousness.

In this framework (illustrated in Figure 2), metacognitive knowledge generates predictions about cognitive performance, while metacognitive monitoring detects errors (mismatches between predicted and actual performance). Metacognitive regulation then adjusts strategies to reduce future errors. This maps onto fundamental neural learning mechanisms: prediction errors drive synaptic modifications that improve future predictions.

However, this theoretical alignment does not establish that metacognitive training specifically enhances neuroplasticity beyond domain-specific learning. A critical empirical question remains: Does explicit training in metacognitive monitoring and regulation produce neural changes beyond those resulting from simply practicing the target cognitive skills?

#### 4.2.2 Problem-solving perspective

An alternative framework views metacognition as domain-general problem-solving processes applied to cognitive tasks (Hulbig, 2023, 2024). From this perspective, metacognitive training teaches systematic approaches to defining learning problems, generating and testing strategies, monitoring outcomes, and iterative refinement—procedures applicable across domains.

This framework suggests that metacognitive training might support transfer of learning across contexts by establishing generalizable self-regulatory procedures. Neural plasticity would result from both domain-specific skill development and the development of more efficient control processes. Whether such training produces dissociable neural changes in control networks versus task-specific regions requires investigation.

#### 4.2.3 Awareness and consolidation

A third possibility is that metacognitive awareness facilitates memory consolidation and skill learning. Explicit awareness of what one has learned and how effectively one performed might enhance hippocampal-mediated memory consolidation and strengthen learning-related synaptic changes. This could explain why metacognitive scaffolding enhances training outcomes (Rueda et al., 2018) even when the primary training targets domain-specific skills.

These frameworks are not mutually exclusive and may capture complementary aspects of how metacognitive processes interface with neural learning mechanisms. Distinguishing among these possibilities requires carefully designed studies manipulating metacognitive training components while measuring both immediate behavioral effects and longer-term neural changes.

### 4.3 Limitations and future directions

Several key areas require further investigation. First, we need more precise methods for assessing individual differences in metacognitive ability and predicting responsiveness to neuroplastic interventions. Understanding which individuals and which conditions benefit most from specific types of metacognitive training will enable personalized intervention approaches.

Second, longitudinal studies tracking metacognitive development and neural changes across the lifespan are essential. Understanding the developmental trajectories of different metacognitive components and critical periods for neuroplastic intervention will optimize timing and intensity of treatments.

Third, research should focus on optimizing the integration of metacognitive training with interventions known to promote neuroplasticity. What combinations of cognitive training, physical exercise, meditation, and pharmacological support produce maximal neural reorganization? What dosing, sequencing, and duration parameters optimize outcomes? These questions are crucial for translating basic science into effective clinical applications.

Fourth, emerging technologies including virtual reality, neurofeedback, and brain stimulation may enhance metacognitive training effects by providing more immediate feedback on neural activity and enabling more targeted interventions. The potential for real-time monitoring and adjustment of metacognitive training based on neural responses represents an exciting frontier.

## 5 Conclusion

The neurological investigation of metacognition holds tremendous potential for addressing neurological differences and optimizing human development. By directing our powers of understanding and problem-solving toward the mechanisms of understanding and problem-solving themselves, we achieve a profound recursion that generates both deeper knowledge and more sophisticated behavioral regulation.

The convergence of metacognition and neuroplasticity research provides a scientifically grounded pathway to neural reorganization with broad implications. Rather than viewing neurological differences as fixed constraints, we can conceptualize them as targets for neuroplastic intervention through metacognitive training. The evidence reviewed here demonstrates that such interventions produce lasting changes: structural brain reorganization, enhanced network connectivity, improved clinical outcomes, and sustained educational gains.

As we continue to uncover the neural foundations of metacognitive ability and refine our understanding of neuroplastic mechanisms, we simultaneously enhance our capacity to develop more effective interventions. This creates a positive feedback loop: better understanding of metacognition-neuroplasticity integration leads to improved interventions, which generate new insights, which further refine our approaches. This iterative process may prove essential for addressing the complex challenges facing individuals with neurological differences and for optimizing human cognitive potential more broadly.

The science of metacognition thus represents not merely an academic pursuit, but a fundamental and practical effort to harness the brain’s capacity for self-directed change. By understanding how metacognitive processes engage neuroplastic mechanisms, we gain powerful tools for promoting long-term skill development, psychological adaptation, and neural reorganization across the spectrum of human experience. The future of neurological intervention may well rest on our ability to effectively integrate metacognitive training with neuroplasticity-promoting approaches, creating personalized pathways for lasting neural and behavioral change.

## Data Availability

The original contributions presented in the study are included in the article/supplementary material, further inquiries can be directed to the corresponding author.
